# 한국 일개 상급종합병원에서 지속적 신대체 요법을 받는 환자의 필터 수명 시간에 영향을 미치는 요인: 횡단적 기술 연구

**DOI:** 10.7586/jkbns.25.064

**Published:** 2025-11-26

**Authors:** Yuri Lee, Juyoung Lee, Inyoung Ji, Minhee Suh

**Affiliations:** 1 Department of Nursing, Inha University Hospital, Incheon, Korea; 1 인하대학교병원 간호본부; 2 School of Nursing, Inha University, Incheon, Korea; 2 인하대학교 간호대학

**Keywords:** Continuous renal replacement therapy, Micropore filters, Clinical alarms, 지속적 신대체 요법, 필터, 알람 관리

## Abstract

**Purpose:**

Although multiple factors influencing filter lifespan during continuous renal replacement therapy (CRRT) have been identified, the impact of alarm management remains underexplored. This study aimed to investigate factors affecting filter lifespan during CRRT, with particular focus on alarm management variables.

**Methods:**

Data were collected from 114 filters used in 57 patients who underwent CRRT at a university hospital in Korea. Patient factors included hematologic parameters; CRRT prescription factors encompassed set-up parameters; and catheter-related factors included catheter type and location. Alarm management factors were defined as the frequency and duration of alarm occurrences per unit of filter operating time. Their influence on filter lifespan was analyzed.

**Results:**

The mean filter lifespan was 31.38 hours. Hematocrit (*p* = .007), alarm duration (*p* < .001), and maximum alarm duration (*p* < .001) were negatively correlated with filter lifespan, whereas CRRT prescription and catheter-related factors showed no significant associations. Multiple regression analysis identified lower hematocrit (*p* = .026) and shorter alarm duration (*p* < .001) as significant predictors of longer filter lifespan.

**Conclusion:**

Alarm duration was the most influential factor affecting filter lifespan, providing empirical evidence of the crucial role nurses play in effective alarm management during CRRT.

## 서론

### 1. 연구의 필요성

지속적 신대체 요법(continuous renal replacement therapy, CRRT)은 신대체 요법 중 하나로, 환자의 혈액을 천천히 체외로 빼내어 반투막 재질의 필터를 통과시키면서 노폐물 제거, 전해질 교정, 염증 물질 제거 등의 치료를 수행하기 때문에[1] 활력징후가 불안정하거나 중증인 중환자에게 적용된다[2]. 지속적 신대체 요법의 적용은 인구 고령화, 장비의 발전 및 확충 등으로 인해 꾸준히 증가하고 있다. 2005년~2007년에 비해 2014년~2016년에 3배 가까이 증가하였으며[3], 2020년 국내 중환자실 현황조사 보고서[4]에 의하면 중환자실 환자의 12.5%가, 해외의 경우 13.9%[5]가 지속적 신대체 요법을 받고 있는 것으로 나타났다.

지속적 신대체 요법에서는 반투막 재질의 필터를 적정기간 동안 충분히 사용하는 것이 중요한데, 필터를 적정 시간 동안 사용하지 못하고 비 계획적으로 필터를 교체하게 되는 경우에는 혈액 소실, 치료 기간 연장, 치료비용 증가 등이 초래되기 때문이다[6]. 따라서 의료진은 지속적 신대체 요법을 받는 환자의 필터 수명 시간을 관리하고 있다[7]. 필터 수명 시간(filter life span)이란 새로운 필터를 적용하여 지속적 신대체 요법을 시작한 후 혈액 응고로 해당 필터를 교체하기까지 소요되는 시간을 의미한다[8]. 적정 필터 수명 시간을 유지하기 위해 의료인들은 다양한 노력을 하고 있으며, 해외에서는 필터 수명 시간에 영향을 미치는 요인들을 다각적으로 연구하고 있다.

필터 수명 시간에 영향을 주는 요인은 다양한 것으로 알려져 있다. 간 부전 환자[9,10], 활성화 부분 트롬보플라스틴 시간(activated partial thromboplastin time)[11] 등이 필터 수명 시간에 영향을 미치는 것으로 보고된 바 있으며, 지속적 신대체 요법의 종류 및 카테터 삽입 위치나 유형[12], 혈류 속도(blood flow rate)[13], 여과분율(filtration fraction)이나 유출량(effluent dose)[14,15]도 필터 수명 시간에 영향을 미치는 것으로 나타났다. 최근의 메타분석 연구[16]에 의하면 항응고제 사용, 헤모글로빈 수치, 필터 압력, 필터 세척 여부, 희석 방법 등이 비계획적 신대체 요법 중단과 관련되는 것으로 보고되었다. 그러나 상반된 결과들[17-19]도 보고되고 있어, 이에 대한 통합적인 분석이 필요하다.

뿐만 아니라, 지속적 신대체 요법 적용 중에는 다양한 이유로 기계의 알람이 울리게 된다. 지속적 신대체 요법 기기에서 알람이 발생하면, 혈류 속도가 느려지거나 멈추게 되며, 혈액의 응고가 진행된다[20]. 이때 혈액 응고의 진행을 최소화하여 필터 수명 시간을 유지하기 위해서 알람을 해결하는 간호사의 역량이 중요하게 여겨진다. 이에 기반하여 선행 연구들에서는 알람의 횟수를 줄이고 알람 해결 시간을 단축시키는 것이 필터 수명 시간을 연장시킬 수 있다는 가정하에 담당 간호사에게 알람 프로토콜을 적용하여 알람 해결률을 높이거나[21], 시뮬레이션 프로그램 적용 및 교육을 통하여 알람 발생률을 줄이려고 시도하였다[22,23]. 그럼에도 불구하고, 이러한 알람 관리 요인이 직접적으로 필터 수명 시간 연장에 영향을 주는지에 대한 실증적인 연구는 거의 없는 실정이다. 따라서 알람 관리 요인이 필터 수명 시간에 영향을 미치는지 확인할 필요가 있다.

따라서 본 연구에서는 필터 수명 시간에 영향을 미치는 요인을 환자 요인, 지속적 신대체 요법 처방 요인, 카테터 요인, 알람 관리 요인으로 나누어 통합적으로 조사하여 분석해 보고자 한다.

### 2. 연구의 목적

본 연구의 목적은 지속적 신대체 요법 적용 시 필터 수명 시간에 영향을 주는 요인을 확인하는 것이다.

1) 지속적 신대체 요법시 필터 수명 시간을 조사한다.

2) 지속적 신대체 요법시 환자 요인, 지속적 신대체 요법 처방 요인, 카테터 요인, 알람 관리 요인을 조사한다.

3) 지속적 신대체 요법시 환자 요인, 지속적 신대체 요법 처방 요인, 카테터 요인, 알람 관리 요인에 따른 필터 수명 시간을 확인한다.

4) 지속적 신대체 요법시 환자 요인, 지속적 신대체 요법 처방 요인, 카테터 요인, 알람 관리 요인이 필터 수명 시간에 주는 영향을 분석한다.

## 연구 방법

### 1. 연구 설계

본 연구는 지속적 신대체 요법 적용 시 필터 수명 시간에 영향을 주는 요인을 확인하는 전향적 서술적 조사연구이다.

### 2. 연구 대상

본 연구는 인하대학교병원에서 2024년 5월부터 12월의 기간 중 지속적 신대체 요법을 시작한 중환자를 총 57명을 대상으로 하였다. 일반적으로 지속적 신대체 요법 시작 후 초기에 여러 요인을 조정하는 과정 동안 혈액 응고로 인한 비계획적 필터 교체가 빈번하게 발생하므로[24,25] 지속적 신대체 요법 시작 후 첫 번째, 두 번째 필터를 적용 중인 환자를 대상으로 하며, AV 600S hemofilter (Fresenius Medical Care, Bad Homburg, Germany)를 사용하는 환자를 연구 대상으로 포함하였다. 환자의 혈압저하, 사망으로 지속적 신대체 요법 치료를 중도 중단한 경우, 낮은 요소 청소율(filtrate urea nitrogen/blood urea nitrogen)으로 인해 필터를 교체한 경우, 신기능 회복으로 지속적 신대체 요법을 중단하였거나 신기능 악화로 재시작한 경우는 연구에서 제외하였다.

**Figure 1.**
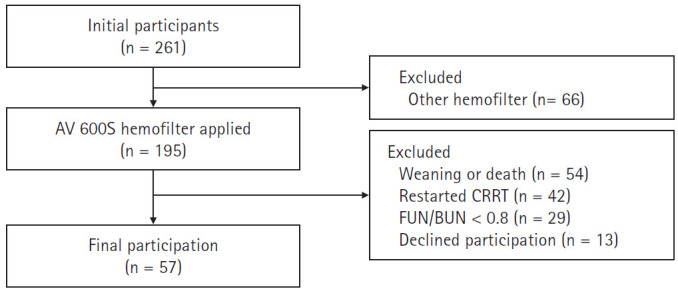
Flow diagram of participant selection. CRRT = Continuous renal replacement therapy; FUN/BUN = Filtrate urea nitrogen/blood urea nitrogen.

2024년 5월부터 12월의 기간 중에 지속적 신대체 요법을 시작한 중환자 총 261명 중 AV 600S hemofilter를 사용한 환자는 195명이었으며. 그중 사망 또는 회복되어 지속적 신대체 요법의 첫 번째 필터만 사용 후 치료가 중단된 대상자 54명, 지속적 신대체 요법을 중단하였다가 재시작한 환자 42명, 낮은 요소 청소율로 인해 필터를 교체한 대상자 29명, 연구 참여를 거부한 대상자 13명이 제외되어 최종적으로 총 57명의 환자가 본 연구에 포함되었으며(Figure 1), 이 환자들이 사용한 1번째 필터와 2번째 필터, 총 114개 필터의 필터 수명 시간을 분석하였다.

본 연구 대상자의 표본 수는 G*power 3.1 program (Heinrich-Heine-Universität, Düsseldorf, Germany)을 활용하여 산정하였다. 필터 수명 시간에 영향을 미치는 요인을 파악하기 위해 회귀분석(multiple linear regression) 통계를 기준으로 유의수준 .05, 중간 정도의 효과 크기 .15, Cohen의 법칙에 따라 검정력 .90, 변수 수 4개를 기준으로 설정하였을 때 최소 표본 수 108개인 것으로 나타났다.

### 3. 연구 도구

#### 1) 필터 수명 시간

필터 수명 시간은 새로운 필터를 적용하여 지속적 신대체 요법을 시작한 후, 혈액 응고 등의 이유로 비계획적으로 해당 필터를 교체하기까지의 시간을 시간 단위로 측정하였다. 비계획적인 필터 교체란 필터 횡단막 압력(transmembrane pressure)이 250 mmHg 이상 지속되거나, 필터 전 압력(prefilter pressure)이 300 mmHg 이상 측정되어 필터 전 압력 높음 알람이 울리거나, 필터 또는 회로관 내에서 심각한 혈액응고가 육안으로 확인되어 필터 교체를 시행한 경우를 말한다.

#### 2) 환자 요인

의무 기록을 토대로 환자 관련 정보를 수집하였고, 환자의 나이, 성별, 지속적 신대체 요법을 시작한 사유, 활성화 부분 트롬보플라스틴 시간, 활성혈액응고시간(activated coagulation time), 적혈구 용적률(hematocrit)을 포함하였다.

#### 3) 지속적 신대체 요법 처방 요인

지속적 신대체 요법의 치료 종류, 항응고제 사용, 평균 혈류 속도(average blood flow rate), 평균 유출량(average effluent dose), 평균 여과분율(average filtration fraction)을 의무 기록을 토대로 자료를 수집하였다.

평균 혈류 속도는 처방에 따라 설정되었고, 필터 1개를 사용하는 동안 적용된 혈류 속도의 평균으로 측정하였으며, 단위는 mL/min이다.

유출량(effluent dose)은 지속적 신대체 요법 치료 과정에서 환자의 체외로 제거된 노폐물과 용질의 총량을 의미하며, 처방된 투석액 유량(dialysate flow rate, DFR), 보충액 유량(replacement flow rate, RFR), 초여과 유량(ultrafiltration rate, UFR)의 합을 환자 체중으로 나눈 값을 말한다. 본 연구에서는 필터를 사용하는 동안의 시간당 평균 유출량을 측정하였으며, 이는 필터 1개를 사용하는 동안 유출량을 필터 수명 시간으로 나누어 산출하였다. 단위는 ml/kg/hr이다.


$$aEffluent\;dose\;\left(mL/kg/hr\right)=\frac{\left(DFR+RFR+UFR\right)\;\left(ml\right)}{체중\left(kg\right)\;x\;필터\;수명\;시간\left(hr\right)}$$

여과분율(filtration fraction)은 필터로 유입되는 혈장수(plasma water)의 유속에 대비하여 초여과를 통해 제거되는 수분의 비율을 의미한다. 본 연구에서는 매시간 여과분율을 모두 합한 후 필터 수명 시간으로 나누어 평균 여과분율을 산출하였다.

#### 4) 카테터 요인

본 연구에서는 의무 기록을 확인하여 카테터 종류와 삽입 위치에 대한 정보를 수집하였다.

#### 5) 알람 관리 요인

지속적 신대체 요법 기기에 저장된 알람 발생 기록을 통하여 알람 내역을 확인하였다. 혈류의 중단이 1 분 이상 지속되면 필터 응고로 진행될 수 있으므로[20] 지속적 신대체 요법의 알람 중 혈류 중단을 유발하는 알람인 유입 압력(access pressure) 알람, 반환 압력(return pressure) 알람, 필터 전 압력 알람, 필터 횡단막 압력 알람이 울린 경우 알람으로 간주하였다. 유입 압력 알람, 반환 압력 알람은 주로 카테터의 꼬임, 위치 이상, 혈관 내 저항 증가와 같은 혈류의 유입과 반환문제로 발생하며, 필터 전 압력 알람, 필터 횡단막 압력 알람은 필터 내 혈전(clotting) 형성이나 필터 막힘(clogging) 등 필터 관련 요인으로 인해 발생한다[26].

알람의 발생은 알람 시작부터 해결까지를 1회로 정의하였으며, 동일 알람이 1분 이내에 다시 발생하지 않을 경우 해당 알람이 해결된 것으로 보았으며, 본 연구에서는 알람 발생 빈도, 알람 지속시간, 최대 알람 지속시간을 수집하였다. 알람 발생 빈도는 1개의 필터를 사용하는 동안 발생한 알람 총횟수를 해당 필터의 수명 시간으로 나누어서 산출하였고, 알람 지속시간은 1개의 필터를 사용하는 동안 발생한 알람 총 지속시간을 해당 필터의 수명 시간으로 나누어, 필터 운용시간 대비 알람 빈도와 지속시간의 상대적 비율을 산출하였다. 최대 알람 지속시간은 필터 1개를 사용하는 동안 가장 길게 지속된 알람의 시간으로 정의하였다.

### 4. 자료 수집

2024년 5월부터 12월의 기간에 진행되었다. 해당 부서 관리자에게 연구 목적을 설명하고 연구 진행 및 자료 수집에 대한 허락을 구하였다. 연구자는 지속적 신대체 요법을 받는 환자가 발생하면 선정 기준에 포함되는지 확인하였고, 해당 부서를 방문하여 대상자에게 연구에 대해 설명하고 참여 동의를 구한 뒤, 해당 환자의 의무 기록, 지속적 신대체 요법 기계의 알람 발생 내역, 필터 수명 시간을 조사하였다. 의식이 명료하지 않거나 진정 상태 등으로 본인의 직접 동의가 어려운 대상자의 경우에는, 보호자에게 연구 목적과 절차를 설명하고 동의를 받은 후 자료를 수집하였다.

### 5. 자료 분석

본 연구에서 수집된 자료는 R 통계 프로그램(version 4.3.1; R Foundation for Statistical Computing, Vienna, Austria)을 이용하여 분석하였다. 연구 변수들의 정규성을 확인하기 위해 왜도와 첨도를 확인한 결과, 알람 관리 요인 변수들은 왜도 ± 2 이상, 첨도 ± 10 이상으로 나타나[27], 비모수 통계를 시행하거나 로그 변환 후 분석에 이용하였으며 구체적인 방법은 다음과 같다.

1) 환자 요인, 지속적 신대체 요법 처방 요인, 카테터 요인, 필터 수명 시간은 실수와 백분율, 평균과 표준편차로 제시하였고, 알람 관리 요인 변수들은 중앙값과 사분위 범위를 제시하였다.

2) 지속적 신대체 요법 시 환자 요인, 지속적 신대체 요법 처방 요인, 카테터 요인과 필터 수명 시간간의 관련성을 분석하기 위해 t-test, ANOVA, Pearson 상관분석을 시행하였고, 알람 관리 요인과 필터 수명 시간간의 관련성을 분석하기 위해 Spearman 상관분석을 이용하여 분석하였다.

3) 지속적 신대체 요법 시 환자 요인, 지속적 신대체 요법 처방 요인, 카테터 요인, 알람 관리 요인이 필터 수명 시간에 미치는 영향을 확인하기 위해 다중 선형 회귀분석을 시행하였다. 다중 선형 회귀분석시, 단변량 분석 결과 필터 수명 시간과 관련성이 *p* < .05를 만족하는 변수들을 독립 변수로 포함하였고, 알람 관리 요인 변수들은 로그 변환하여 분석하였다. 독립 변수는 동시 입력 방법으로 회귀분석에 투입하였다.

### 6. 윤리적 고려

본 연구는 인하대학교병원 기관생명윤리 위원회의 승인을 받았다(IRB No. 2024-04-026-003). 대상자에게 연구 목적과 절차를 설명하고 동의서를 받았으며, 자발적 참여 의사를 밝힌 대상자에 한해서 연구에 참여하였다. 대상자가 진정 약물을 투여받고 있거나 의식이 명료하지 않은 등 대상자에게 동의서를 받을 수 없는 경우에는 보호자에게 동의서를 받았다.

수집된 자료는 개인 정보 보호를 위해 익명화 및 코드화하였으며, 보안 유지를 위해 컴퓨터 암호화된 파일에 저장하였다. 또한 동의서는 종이봉투에 담아 자물쇠가 있는 사물함에 보관하였다. 본 연구의 자료는 연구자, 공동 연구자만 열람할 수 있었으며, 수집된 자료는 연구 목적 이외에 활용되지 않았다. 또한 연구의 모든 과정에서 어떠한 사유로든 자료를 배포하거나 유출되지 않도록 하였으며 연구 종료를 기준으로 3년이 지난 후에 복원 불가능한 방법으로 폐기할 예정이다.

## 연구 결과

### 1. 연구 대상자 특성 및 지속적 신대체 요법 필터 수명 시간

대상자의 성별은 남자가 39명(68.4%), 여자가 18명(31.6%)였으며 연령은 60~79세가 32명(56.1%)으로 가장 많았다(Table 1). 지속적 신대체 요법 시작 사유로는 패혈증이 33명(57.9%)로 가장 많았다. 총 57명의 대상자의 지속적 신대체 요법 치료에 사용된 총 114개의 필터의 평균 수명 시간은 31.38 ± 16.33 시간이었으며 1번째 필터의 평균 수명 시간은 30.85 ± 16.22 시간, 2번째 필터의 평균 수명 시간은 31.91 ± 16.56 시간으로 2번째 필터에서 수명 시간이 다소 길어지는 경향이 있었으나 통계적으로 유의한 차이를 보이지 않았다.

### 2. 연구 변수의 기술통계

환자 요인인 활성화 부분 트롬보플라스틴 시간은 52.04 ± 14.70초, 활성혈액응고시간은 150.20 ± 29.84초, 적혈구용적률은 29.02 ± 7.17%였으며, 처방 요인으로 조사한 요법 종류는 지속적 정맥-정맥 혈액투석여과(continuous veno-venous hemodiafiltration, CVVHDF)가 92.1%, 지속적 정맥-정맥 혈액투석(continuous veno-venous hemodialysis, CVVHD)가 7.9%였다(Table 2). 항응고제를 사용하지 않은 대상자는 31.6%, Nafamostat mesylate를 사용한 대상자는 61.4%, Heparin을 사용한 대상자는 7.0%였으며, 평균 혈류 속도는 150.70 ± 8.80 mL/min, 평균 유출량은 35.16 ± 5.04 mL/kg/hr, 평균 여과분율은 13.95 ± 4.55% 였다. 카테터 종류는 비터널식 혈액투석도관(non-tunneled central venous dialysis catheter)가 77.2%로 가장 많았고 터널식 혈액투석도관(tunneled central venous dialysis catheter)가 18.4%, 체외막산소공급(extracorporeal membrane oxygenation) 연결이 4.4% 였다. 삽입 부위는 대퇴부가 62.3%로 가장 많았고 경정맥은 33.3% 였다.

알람 관리 요인인 알람 발생 빈도의 중앙값은 0.14 (interquartile range, 0 .05~0.44)회, 알람 지속시간의 중앙값은 6.25 (0.96~20.47)초, 최대 알람 지속시간의 중앙값은 76.50 (19~147.20)초였다.

### 3. 필터 수명 시간 관련 요인

필터 수명 시간과 연구 변수 간의 단변량 분석 결과, 필터 수명 시간은 환자의 혈액 검사 중 적혈구용적률(*r* = −.25, *p* = .007)과 유의한 관련성을 보였으나, 활성화 부분 트롬보플라스틴 시간, 활성혈액응고시간과는 유의한 연관성을 보이지 않았다(Table 2). 처방 요인인 요법 종류, 항응고제 사용, 평균 혈류 속도, 평균 유출량, 평균 여과분율, 카테터 요인인 카테터 종류 및 삽입 위치는 모두 필터 수명 시간과 유의한 연관성을 보이지 않았다.

알람 관리 요인인 알람 발생 빈도(*r* = −.50, *p* < .001), 알람 지속시간(*r* = −.54, *p* < .001), 최대 알람 지속시간 (*r* = −.33, *p* < .001)이 모두 필터 수명 시간과 유의한 음의 상관관계를 보였다.

필터 수명 시간에 영향을 미치는 요인을 살펴보기 위하여 다중 선형 회귀분석을 시행하였다 (Table 3). Durbin-Watson 지수는 2.20으로 종속변수들이 자기상관성 없이 독립적이었으며, 공차(tolerance)는 0.101~0.953 로 0.1 이상이었고, 분산 팽창지수(variance inflation factor)는 1.050~9.864 로 10 이하이므로 다중 공선성의 문제는 없는 것으로 나타났다.

다중 선형회귀 분석 결과, 적혈구용적률이 낮을수록(*β* = −0.16, *p* = .026), 알람 지속시간이 짧을수록(*β* = −1.68, *p* < .001), 알람 발생 빈도가 높을수록(*β* = 0.49, *p* < .001), 최대 알람 지속시간은 길어질수록(*β* = 0.92, *p* < .001) 필터 수명 시간이 유의하게 증가되는 것으로 나타났다. 이 회귀모형의 설명력은 47.0%였고, 모형은 유의하였다(F = 26.24, *p* < .001).

## 논의

본 연구는 지속적 신대체 요법 적용 시 필터 수명 시간에 영향을 미치는 요인을 확인하고자 시행하였다.

본 연구의 필터 수명 시간의 평균값은 31.38시간으로 나타났다. Zhao 등[28]의 연구에서 필터 수명 시간은 중앙값이 39.22시간으로 본 연구의 필터 수명 시간보다 길었다. 일반적으로 필터 수명 시간은 최대 72시간이지만, 국내 임상에서는 필터 흡착 능력 효과와 체외 순환에 의한 감염의 위험성을 고려하여 48시간을 기준으로 필터가 교체되도록 권고된다. 이에 따라 본 연구에서는 필터를 48시간 초과하여 사용한 비율이 9.65%로, Zhao 등[28]의 연구에서의 37.76%보다 낮았기 때문에 이러한 차이가 나타났을 수 있다. 국내 연구인 Heo 등[29]에서는 평균 필터 수명 시간이 36.3 시간으로 보고되어 본 연구와 비교해 다소 길거나 유사하였다. 다만 Heo 등[29]의 연구는 항응고제를 사용하는 환자만을 포함하여 전반적인 필터 사용시간이 길었던 것으로 보인다.

본 연구에서 환자 요인 중 활성화 부분 트롬보플라스틴 시간은 필터 수명 시간과 유의한 상관성을 보이지 않았다. 이는 선행연구[30]에서 활성화 부분 트롬보플라스틴 시간이 낮을수록 필터 수명 시간이 유의하게 단축되었다는 결과와 상이하였다. 선행연구[30]에서는 활성화 부분 트롬보플라스틴 시간을 44.2초 기준으로 제시하여, 이보다 낮은 경우 필터 수명 시간이 단축되는 것으로 보고하였는데, 본 연구 대상자들에서 활성화 부분 트롬보플라스틴 시간이 52.04 초로 전반적으로 연장되어 있었기 때문에 필터 수명 시간에 유의한 영향을 보이지 않았던 것으로 생각된다. 한편, 적혈구용적률 수치는 다중회귀분석에서도 필터 수명 시간에 유의한 영향을 주는 독립적인 예측 인자로 확인되었으며, 이는 선행연구[30]의 연구 결과와 일치하는 것으로, 높은 적혈구용적률 수치는 필터 수명 시간 단축에 주요한 영향을 미치는 것으로 추측할 수 있었다.

본 연구에서 평균 여과분율, 지속적 신대체 요법의 종류, 평균 유출량 등의 처방 요인은 필터 수명 시간과 유의한 상관성을 나타내지 않았다. 일반적으로 필터의 조기 막힘을 예방하기 위해 여과분율을 25% 이하로 유지하도록 권고되고 있는 것[14]과 비교했을 때에는 다소 차이를 보였으나, 한편으로는 여과분율이 높을수록 필터 수명 시간이 단축된다는 이론의 임상적인 근거는 부족하다는 주장[31]과는 유사하다. 본 연구에서 평균 여과분율이 13.95%로 권고 수준인 25%보다 훨씬 낮게 유지되었는데, 이러한 경우에는 여과분율이 필터 수명 시간에 유의한 영향을 미치지 않았던 것으로 볼 수 있겠다. 비슷한 맥락에서 CVVHDF 요법의 경우에도 CVVHD 요법에 비해 여과분율이 높기 때문에 필터 수명 시간이 짧아질 수 있을 것으로 추측할 수 있는데, 본 연구에서는 치료 요법의 종류도 유의하지 않은 것으로 나타났고, 최근 선행 연구 결과[32]에서도 신대체 요법의 종류가 필터 수명 시간에 유의한 영향을 미치지 않아 본 연구결과와 유사하였다.

본 연구에서 비록 통계적으로 유의하지는 않았으나 필터 수명 시간과 평균 혈류 속도가 오히려 부적인 상관성을 보였다. 이러한 결과는 본 연구 대상자의 대부분이 패혈증으로 인한 저혈압 상태여서 초기 혈류 속도가 150 mL/min 내외로, Dunn과 Sriram [33]이 제시한 최적 혈류 속도인 250~300 mL/min보다 기본적으로 낮게 설정된 것과 관련될 수 있다. 이로 인해 본 연구 대상자들의 필터 응고가 진행되는 것으로 보이는 경우에는 의료진이 혈류 속도를 증가시켜 필터 내 혈액 정체를 줄이고, 여과분율을 낮춤으로써 필터 수명 시간을 연장시키려는 경향이 있었다. 그러나 이러한 점이 오히려 평균 혈류 속도가 높은 경우 필터 수명 시간이 낮아지는 결과로 나타났을 수 있다.

본 연구에서 알람 관련 요인 지표들과 필터 수명 시간의 상관성을 단변량 분석한 결과, 알람 빈도가 잦고, 알람 지속시간이나 최대 알람 지속시간이 길수록 유의하게 필터 수명 시간이 단축되는 것으로 확인되었다. 뿐만 아니라, 회귀분석 결과에서는 알람 지속시간이 필터 수명 시간에 가장 큰 영향력을 보이는 것으로 나타났다. 이는 질 관리가 된 지속적 신대체 요법 간호를 받은 군에서 지속적 신대체 요법이 효율적으로 적용되었다는 연구 결과[34]와 유사한 맥락으로 볼 수 있으나, Zhao 등[28]의 연구에서 치료 중단 시간과 필터 수명 시간이 유의한 관련성이 없었던 것과는 상이하였다. Zhao 등[28]의 연구에서는 치료 중단 시간 지표에 혈액 펌프가 멈추지 않는 알람 및 상황(용액 백 교체, 검사, 시술 등)을 포함하였기 때문에 알람 지속시간의 독립적인 효과가 희석되었을 가능성이 있다. 따라서, 치료 중단 자체보다는 혈액 펌프를 멈추게 하는 알람의 총 지속시간 증가가 필터 수명 시간 단축에 중요한 영향을 미칠 수 있다고 추측해 볼 수 있다. 한편, 본 연구의 회귀분석 결과에서는 알람 발생 빈도나 최대 알람 지속시간의 증가는 오히려 필터 수명 시간 연장과 관련이 있는 것으로 나타났는데, 최대 알람 지속시간은 필터 전체의 알람 관리 양상을 반영한 다기보다는, 특정 시점의 상황을 보여주는 지표였기 때문으로 해석된다. 특히 필터를 장시간 사용한 시점에서 갑작스럽게 필터 응고관련 알람이 발생하고 혈액을 반환하기 위한 노력하는 과정에서 같은 알람이 반복적으로 발생한 시간이 포함되어 있으며, 이러한 임상적 특성이 최대 알람 지속시간이 필터 수명 시간과 정적인 관련성을 보이게 한 요인으로 판단된다. 따라서, 알람의 발생 빈도 자체나 최대 알람 지속시간이 필터 응고로 이어지기 보다는 알람 발생 후 신속히 대응하여 전체적인 알람 지속시간을 줄이는 것이 필터 수명 시간이 조기에 종료되는 것을 예방할 수 있음을 추측해 볼 수 있다.

본 연구의 제한점은 다음과 같다. 첫째, 본 연구는 단일 기관에서 대상자 동질성을 위해 단일 회사 장비를 대상으로 시행되었으며 연구 대상자의 수가 적어 연구결과를 일반화하기에는 제한점이 있다. 그러므로 향후 다기관, 대규모 전향적 연구를 통한 추가 연구가 필요하다. 둘째, 본 연구에서는 알람이 발생한 근본적인 원인(카테터 기능저하, 혈압저하, 회로 응고 진행 등)을 조사하지 않았다. 따라서 알람 발생의 임상적 원인까지 함께 분석하는 후속 연구가 필요하다.

그럼에도 불구하고, 본 연구는 국내에서 지속적 신대체 요법 적용 중 발생하는 알람 내역을 직접 수집하여 필터 수명 시간과의 상관관계를 분석한 최초의 연구로, 필터 중지 관련 알람의 총 지속시간이 길어질수록 필터 수명 시간이 단축됨을 확인함으로써, 지속적 신대체 요법 시행 중 발생하는 알람 관리의 중요성을 확인할 수 있었다는 점에서 의의가 있다.

## 결론

본 연구는 지속적 신대체 요법 적용 시 필터 수명 시간에 영향을 미치는 요인을 확인하고자 시행되었다. 본 연구 결과, 평균 필터 수명 시간은 약 31시간이었으며, 환자 요인 중 적혈구용적률이 높을수록 필터 수명 시간이 단축되는 것으로 나타났다. 또한 알람 관리 요인 중 증가된 알람 지속시간은 필터 수명 시간을 단축시키는 가장 큰 영향 요인으로 확인되었으나, 알람 발생 빈도 및 최대 알람 지속시간은 정적인 방향성을 보여 알람 발생 후 신속히 대응하여 전체적인 알람 시간을 줄이는 것이 필터 수명 시간 연장에 중요함을 시사하였다. 지속적 신대체 요법의 처방 요인이나 카테터 요인들은 필터 수명 시간과 유의한 관련이 없었는데, 이러한 요인들은 임상적으로 권장되는 범위 내에서 적절히 유지되는 경우, 필터 수명 시간에 유의미한 영향을 미치지 않는 것으로 보인다.

본 연구 결과는 지속적 신대체 요법 수행 시 알람 관리가 중요함을 실증적으로 제시하였으며, 이를 통해 지속적 신대체 요법시 간호사의 알람 관리의 중요성에 대한 이론적 근거를 제공하였다는데 그 의의가 있다. 간호사가 신대체 요법 적용 환자 간호시 비계획적 필터 교체로 이어질 수 있는 알람을 선별하고, 해당 알람이 발생했을 때에 신속히 대처한다면 불필요한 의료 자원 소모 예방 및 의료비 절감에 기여할 수 있을 것으로 생각된다.

## Figures and Tables

**Table 1.** Characteristics of Patients and Filter Lifespan (N = 57)

Characteristics		n (%) or M ± SD	Min~Max
Sex	Men	39 (68.4)	
	Women	18 (31.6)	
Age (years)	Under 40	4 (7.0)	
	40 ~ 59	12 (21.1)	
	60 ~ 79	32 (56.1)	
	80 and over	9 (15.8)	
Reason for initiating CRRT	Sepsis	33 (57.9)	
	Nephrotoxin	4 (7.0)	
	Dehydration	1 (1.8)	
	GI bleeding	2 (3.5)	
	Postoperative AKI	1 (1.8)	
	Hepatorenal	1 (1.8)	
	Cardiorenal	8 (14.0)	
	Other (ESRD, IICP)	7 (12.2)	
Filter lifespan (hours)	Total	31.38 ± 16.33	4.00~57.00
	First filter	30.85 ± 16.22	6.50~57.00
	Second filter	31.91 ± 16.56	4.00~51.00

M = Mean; SD = Standard deviation; CRRT = Continuous renal replacement therapy; GI = Gastrointestinal; AKI = Acute kidney injury; ESRD = End-stage renal disease; IICP = Increased intracranial pressure; Min = Minimum; Max = Maximum.

**Table 2.** Characteristics of Study Variables and Their Relationships with Filter Lifespan (N = 114)

Characteristics		n (%) or M± SD	Filter lifespan (hours)	F/t or r	*p*
			M ± SD		
Patient factors	aPTT (second)	52.04 ± 14.70		.14^†^	.152
	ACT (second)	150.20 ± 29.84		.14^†^	.253
	Hct (%)	29.02 ± 7.17		−.25	.007
Treatment type	CVVHDF	105 (92.1)	31.90 ± 16.20	1.14^‡^	.257
	CVVHD	9 (7.9)	25.40 ± 18.20		
CRRT prescription	aBFR (mL/min)	150.70 ± 8.80		−.04	.691
	aED (mL/kg/hr)	35.16 ± 5.04		.04^†^	.708
	aFF (%)	13.95 ± 4.55		−.05	.637
Anticoagulation	None	36 (31.6)	31.60 ± 16.20	0.45	.642
	Nafamostat mesylate	70 (61.4)	30.70 ± 16.80		
	Heparin	8 (7.0)	36.40 ± 12.50		
Type of catheter	Non-tunneled central venous dialysis catheter	88 (77.2)	30.40 ± 16.60	2.09	.151
	Tunneled central venous dialysis catheter	21 (18.4)	33.50 ± 16.10		
	ECMO cannulation	5 (4.4)	40.40 ± 8.65		
Site of catheter insertion	Femoral vein	71 (62.3)	30.80 ± 16.70	0.80	.451
	Jugular vein	38 (33.3)	31.20 ± 16.30		
	ECMO cannulation	5 (4.4)	40.40 ± 8.65		
Alarm management factor	Alarm frequency	0.14 (0.05~0.44)^§^		−.50^∥^	< .001
	Alarm duration	6.25 (0.96~20.47)^§^		−.54^∥^	< .001
	Maximum alarm duration (second)	76.50 (19.0~147.20)^§^		−.33^∥^	< .001

M = Mean; SD = Standard deviation; aPTT = Activated partial thromboplastin time; ACT = Activated coagulation time; Hct = Hematocrit; CVVHDF = Continuous veno-venous hemodiafiltration; CVVHD = Continuous veno-venous hemodialysis; aBFR = Average blood flow rate; aED = Average effluent dose; aFF = Average filtration fraction; ECMO= Extracorporeal membrane oxygenation.

† Pearson r;

‡ t-test;

§ Median (IQR);

∥ r from Spearman rank correlation analysis.

**Table 3.** Multiple Regression Analysis of Factors Influencing Filter Lifespan (N = 114)

Variable	B	SE	β	t	p
Constant	37.78	5.42		6.97	< .001
Patient factor					
Hct (%)	−0.36	0.16	−0.16	−2.26	.026
Alarm management factor					
Ln alarm frequency	28.98	7.57	0.49	3.83	< .001
Ln alarm duration	−19.84	2.54	−1.68	−7.83	< .001
Ln maximum alarm duration	9.14	1.54	0.92	5.92	< .001
Adjusted R² = .47, F = 26.24, p < .001					

SE = Standard error; Hct = Hematocrit; Ln = Natural log-transformed.

## Data Availability

The participants of this study did not give written consent for their data to be shared publicly or with third party, so due to the sensitive nature of the research supporting data is not available.
